# Acupuncture for cancer pain: an evidence-based clinical practice guideline

**DOI:** 10.1186/s13020-021-00558-4

**Published:** 2022-01-05

**Authors:** Long Ge, Qi Wang, Yihan He, Darong Wu, Qi Zhou, Nenggui Xu, Kehu Yang, Yaolong Chen, Anthony Lin Zhang, Haiqing Hua, Jinchang Huang, Ka-Kit Hui, Fanrong Liang, Linpeng Wang, Bin Xu, Yufei Yang, Weimin Zhang, Baixiao Zhao, Bing Zhu, Xinfeng Guo, Charlie Changli Xue, Haibo Zhang, International Trustworthy traditional Chinese Medicine Recommendations (TCM Recs) Working Group, Charlie Changli Xue, Haibo Zhang, Xinfeng Guo, Darong Wu, Kehu Yang, Yaolong Chen, Long Ge, Runsen He, Haiqing Hua, Jinchang Huang, Ka-Kit Hui, Fanrong Liang, Zuodi Pan, Linpeng Wang, Bin Xu, Nenggui Xu, Yufei Yang, Anthony Lin Zhang, Weimin Zhang, Baixiao Zhao, Bing Zhu, Qi Wang, Yihan He, Qi Zhou, Lihong Yang, Shaonan Liu, Jieyun Li, Liangying Hou, Qian Zhang, Honghao Lai, Xueling Chen, Xueyi Deng, Fuqin Kang

**Affiliations:** 1 Department of Social Medicine and Health Management, School of Public Health, Lanzhou University, Lanzhou, China; 2 Evidence Based Social Science Research Centre, School of Public Health, Lanzhou University, Lanzhou, China; 3 WHO Collaborating Center for Guideline Implementation and Knowledge Translation, Lanzhou, China; 4 Chinese GRADE Centre, Lanzhou University, Lanzhou, China; 5 Guangdong Provincial Hospital of Chinese Medicine, Guangzhou, China; 6 The Second Affiliated Hospital of Guangzhou University of Chinese Medicine, Guangzhou, China; 7 Guangdong Provincial Academy of Chinese Medical Sciences, Guangzhou, China; 8 Evidence-Based Medicine Center, School of Basic Medical Sciences, Lanzhou University, Lanzhou, China; 9 South China Research Center for Acupuncture and Moxibustion, Medical College of Acu-Moxi and Rehabilitation, Guangzhou University of Chinese Medicine, Guangzhou, China; 10 Key Laboratory of Evidence Based Medicine and Knowledge Translation of Gansu Province, Lanzhou, China; 11 China-Australia International Research Centre for Chinese Medicine, School of Health and Biomedical Sciences, RMIT University, Melbourne, Victoria Australia; 12 Oncology Department of Bayi Hospital, Nanjing University of Chinese Medicine, Nanjing, China; 13 The Third Affiliated Hospital, Beijing University of Chinese Medicine, Beijing, China; 14 Center for East-West Medicine, Department of Medicine, David Geffen School of Medicine, University of California, Los Angeles, Santa Monica, USA; 15 Chengdu University of Traditional Chinese Medicine, Chengdu, China; 16 Beijing Hospital of Traditional Chinese Medicine, Capital Medical University, Beijing, China; 17 Key Laboratory of Acupuncture and Medicine Research of Ministry of Education, Nanjing University of Chinese Medicine, Nanjing, China; 18 Department of Oncology, Xiyuan Hospital, China Academy of Chinese Medical Sciences, Beijing, China; 19 Department of Oncology, Southern Theater Command General Hospital of PLA, Guangzhou, China; 20 School of Traditional Chinese Medicine, Beijing University of Chinese Medicine, Beijing, China; 21 Institute of Acupuncture and Moxibustion, China Academy of Chinese Medicine Sciences, Beijing, China

**Keywords:** Acupuncture, Cancer pain, Practice guideline, Evidence-based practice

## Abstract

**Background:**

This study aims to develop an evidence-based clinical practice guideline of acupuncture in the treatment of patients with moderate and severe cancer pain.

**Methods:**

The development of this guideline was triggered by a systematic review published in *JAMA Oncology in 2020*. We searched databases and websites for evidence on patient preferences and values, and other resources of using acupuncture for treatment of cancer pain. Recommendations were developed through a *Delphi* consensus of an international multidisciplinary panel including 13 western medicine oncologists, Chinese medicine/acupuncture clinical practitioners, and two patient representatives. The certainty of evidence, patient preferences and values, resources, and other factors were fully considered in formulating the recommendations. The Grading of Recommendations Assessment, Development, and Evaluation (GRADE) approach was employed to rate the certainty of evidence and the strength of recommendations.

**Results:**

The guideline proposed three recommendations: (1) a strong recommendation for the treatment of acupuncture rather than no treatment to relieve pain in patients with moderate to severe cancer pain; (2) a weak recommendation for the combination treatments with acupuncture/acupressure to reduce pain intensity, decrease the opioid dose, and alleviate opioid-related side effects in moderate to severe cancer pain patients who are using analgesics; and (3) a strong recommendation for acupuncture in breast cancer patients to relieve their aromatase inhibitor-induced arthralgia.

**Conclusion:**

This proposed guideline provides recommendations for the management of patients with cancer pain. The small sample sizes of evidence limit the strength of the recommendations and highlights the need for additional research.

**Supplementary Information:**

The online version contains supplementary material available at 10.1186/s13020-021-00558-4.

## Background

Cancer is the second leading cause of death globally with an estimated mortality of 10.0 million in 2020 [1, 2]. Pain is one of the most common symptoms in cancer patients, in particular, over 70% of individuals with advanced cancer suffer from moderate to severe pain (Numerical rating scale, NRS ≥ 4) [3, 4].

The World Health Organization (WHO) provides recommendations on pharmacologic and radiotherapeutic management of cancer pain, emphasizing the appropriate application of opioids [5]. However, the opioid crisis [6] has exacerbated the challenges of pain management and highlights a need for nonpharmacological treatments [7]. Acupuncture, as the most common method of traditional Chinese medicine in physical intervention, has been widely used for the control of chronic pain [8]. A recent systematic review and meta-analysis demonstrated that acupuncture was significantly associated with reduced cancer pain and can decrease the use of analgesics with moderate certainty of evidence [9]. Approximately one in 10 cancer survivors have used acupuncture in the United States [10]. Given the benefit, clinical urgency, easy public accessibility, relatively high social acceptance and low cost [11], it is necessary to develop a trustworthy guidance to guide clinical practice of acupuncture for cancer pain management.

Currently, several guidelines of the clinical management of cancer pain are available. Among them, the guidelines from European Oncology Nursing Society (EONS) and National Comprehensive Cancer Network (NCCN) [12, 13] suggest the use of acupuncture for managing cancer pain (Box 1). However, both of them do not provide the practical recommendations for acupuncture practice, which limits the application of acupuncture in clinical cancer pain management. Therefore, a specific guideline with detailed practical points is needed to promote the dissemination and implementation of acupuncture in clinical practice, particularly in the field of oncology [14].

**Box 1:** Current international guidelines on acupuncture for cancer pain

Guidelines	Recommendations
European Oncology Nursing Society breakthrough cancer pain guidelines, 2014	Suggest acupuncture as a complementary strategy for cancer pain (unclear strength of recommendation)
NCCN guideline: Adult Cancer Pain, Version 2.2021, 2021	Suggest acupuncture as a physical intervention for cancer pain (category 2A)

The international Trustworthy traditional Chinese Medicine Recommendations (TCM Recs) Working Group, an international team aimed to produce rigorous evidence-based traditional Chinese medicine recommendations adhering to the trustworthy standards [15], has recruited an international multidisciplinary panel to develop this guideline. This guideline was mainly based on the systematic review published in *JAMA Oncology* [9]. Meanwhile, the working group also systematically reviewed the studies of patient preferences and values, and explored the rule of acupuncture point selection for cancer pain by literature review and *Delphi* method [16, 17].

This guideline is intended for all hospitals and community healthcare services, as well as Chinese medicine physicians, physicians of integrated traditional Chinese and western medicine, clinicians, rehabilitation physicians, acupuncturists, and related researchers. The target population is cancer patients with moderate to severe pain.

## Methods

**Fig. 1:**
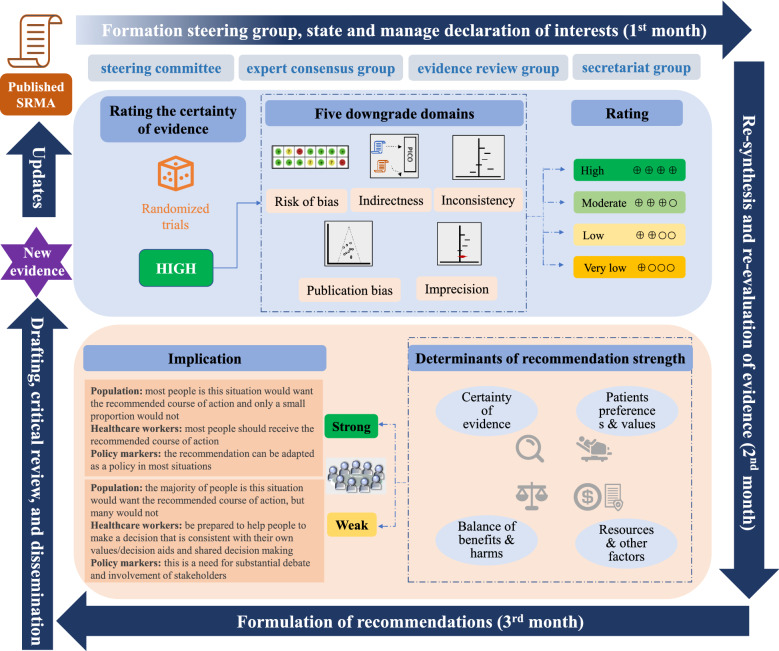
Rapid development process of TCM Recs based on a published systematic review

The design and development of this guideline are in accordance with the “World Health Organization Handbook for Guideline Development” [18]. The guideline was reported in accordance with the Reporting Items for Practice Guidelines in Healthcare (RIGHT) statement [19]. Appraisal of Guidelines for Research and Evaluation II (AGREE II) and Institute of Medicine (IOM) were consulted to ensure the quality of the guideline [15, 20]. The development process of recommendations is summarized in Fig. 1.

### Panel constitution

The guideline panel included representatives from the fields of western medicine, Chinese medicine/acupuncture, guideline and systematic review methodology, as well as two patients. The working group consisted of four subgroups including a steering committee, an expert consensus group, an evidence review group, and an academic secretariat group. The professional fields of the steering committee and the expert consensus group are shown in Additional file 1: Appendix S1. All members of the panel completed a disclosure form, which required disclosure of financial and other interests, including relationships with commercial entities. None of the panel members stated any financial conflict of interest. The members who had academic conflicts of interest (the core authors of *JAMA Oncology* systematic review) did not participate in voting on the recommendations (Additional file 1: Appendix S2).

### Identification of clinical questions

Based on the systematic review published in *JAMA Oncology* [9], in accordance with the Patients, Intervention, Control and Outcomes (PICO) framework, the secretariat group drafted clinical questions. Then, the steering committee conducted an online face-to-face discussion through Microsoft Team meeting, and initially identified five clinical questions: (1) with regard to pain alleviation for cancer patients, what are the effects of acupuncture treatment? (2) For cancer patients using analgesics for pain control, what are the effects of acupuncture/acupressure when combined with analgesics? (3) What are the differences of different acupuncture methods in pain alleviation for cancer patients? (4) What are the acupoints selection, manipulation and course of acupuncture /acupressure treatment for cancer pain? (5) With regard to breast cancer patients with aromatase inhibitor-induced arthralgia, what are the effects of acupuncture treatment? We invited all experts of the consensus group to vote the rationality those five clinical questions. Finally, we decided to describe the third and the fourth clinical questions under the description of the first clinical question as the recommendation statement.

### Synthesis and evaluation of evidence

The evidence review group closely collaborated with the core authors of the *JAMA Oncology* systematic review. Using the studies included in the systematic review, we re-performed data synthesis and certainty of evidence assessment based on the clinical questions. Data synthesis was performed using Review Manager software (RevMan, Version 5.3, Copenhagen: The Nordic Cochrane Centre, The Cochrane Collaboration, 2014). We rated the certainty of evidence for each outcome using GRADE approach [21, 22], which classifies evidence as high, moderate, low, or very low certainty (Additional file 1: Appendix S3). The starting point for certainty for randomized controlled trials (RCTs) is high, but can be rated down based on serious limitations in risk of bias, imprecision, inconsistency (heterogeneity), indirectness, and publication bias. We used minimally important differences (MIDs) to assess the imprecision of summary effect estimates based on the Initiative on Methods, Measurement, and Pain Assessment in Clinical Trials (IMMPACT) recommendations [23].

We adopted literature review and *Delphi* method to explore the rule of acupuncture point selection for cancer pain [24]. We searched Chinese and English databases to include clinical studies on the treatment using acupuncture for cancer pain. We included 28 studies and extracted acupoints and selection principles adopted in the included clinical studies. The questionnaire was designed according to the literature review results, and two rounds of *Delphi* survey were conducted among 34 Chinese and international experts in the field of acupuncture and cancer.

### Patient preferences and values

We searched eight databases from the inception to June 2020 to include primary quantitative (e.g. cross-sectional surveys), qualitative (e.g. interviews, focus groups), and mixed method studies on the preferences and values of patients with cancer pain. Quantitative studies used an adapted version of the GRADE approach to assess risk of bias of studies [25] and qualitative studies used the Critical Appraisal Skills Programme qualitative research checklist [26]. Teams of two independently performed titles and abstracts screening, full-text review, data extraction, and risk of bias assessment. Any conflict was resolved by consensus. Details on search and screening can be found in Additional file 1: Appendix S4. We finally included 8 studies involved 2505 patients with cancer pain. We did not find any published evidence of acupressure addressing patient values and preferences. The results were analyzed and taken into account when developing evidence-based recommendations. Meanwhile, we also included two patients, who have used acupuncture for the treatment of their cancer pain, to participate in voting the recommendations.

### Formulation of recommendations

We used GRADE approach to formulate the recommendations, and categorized the strength of recommendations as “strong” and “weak” (sometime guidelines may use terms such as “conditional” or “discretionary” instead of weak), by comprehensively considering the balance of benefits and harms, the certainty of evidence, the patient preferences and values, resources, and other factors [21, 22]. We searched PubMed and Chinese National Knowledge Infrastructure databases to identify studies on the accessibility, acceptability, and policy support for acupuncture or acupressure.

We used *Delphi* method to reach consensus for all recommendations. The consensus was reached when more than 70% experts agreed with the strength and direction of recommendation. We obtained the feedback regarding the recommendation from 2 patients and 12 experts (one expert did not vote). The acupuncturist first explained to patients the content that needs consensus, and then the patients independently complete the consensus voting on recommendations.

In the first round of *Delphi* survey, all recommendations reached a consensus. Thirteen of 14 experts voted for recommending the treatment of acupuncture to relieve pain and suggesting a combination treatment of acupuncture/acupressure and analgesics to relieve pain and reduce opioid dose in patients with cancer pain. Ten of 14 experts voted for suggesting the treatment of acupuncture rather than no treatment to relieve pain in breast cancer patients with aromatase inhibitor-induced arthralgia. The consensus was also reached for the acupuncture point selection, manipulation and course. The details are shown in Additional file 1: Appendix S5.

### Drafting, critical review, and dissemination

The guideline was drafted by the academic secretariat. After an internal review, a document was developed for open discussion. After receiving feedbacks from experts, the guideline was finalized by another round of peer review facilitated by the journal editor. The guideline will be disseminated by peer-reviewed academic journals, presented at conferences, and promoted in public media outlets such as *WeChat* and *Facebook*.

### Updates

As new evidence is published, the TCM Recommendations group will assess the new evidence and make a judgment on the extent that it would be expected to alter the recommendation. Updated guideline will follow the Checklist for the Reporting of Updated Guidelines (CheckUp) [27].

## Recommendations

**Fig. 2:**
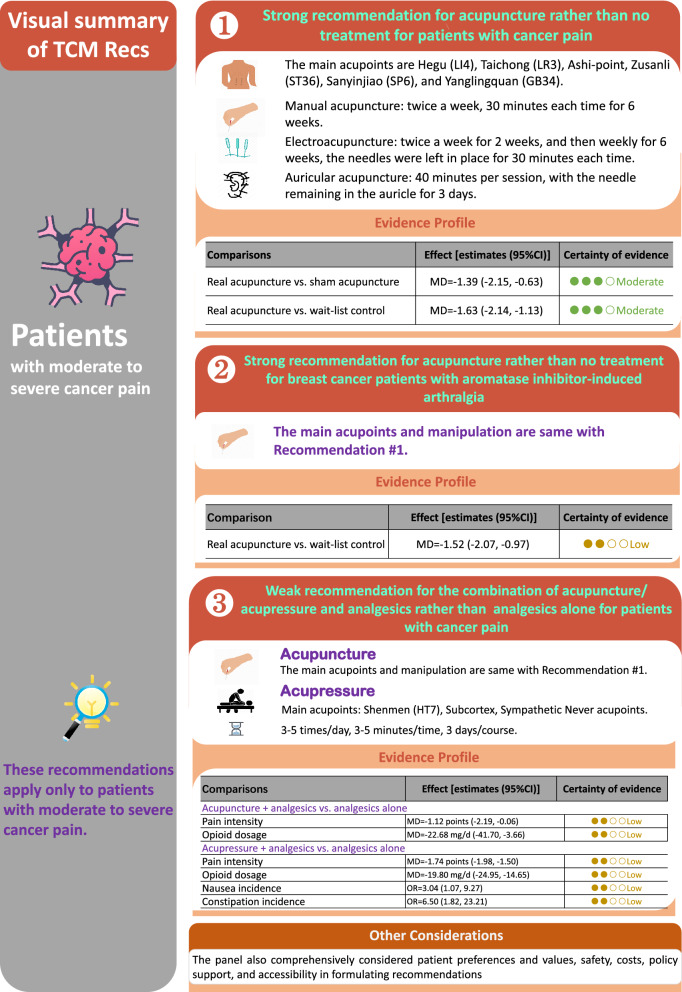
Visual summary of recommendations

The recommendations are summarized in Fig. 2.

### Clinical question 1: With regard to pain alleviation for cancer patients, what are the effects of acupuncture treatment?

#### Recommendation 1

We recommend the treatment of acupuncture rather than no treatment to relieve pain in patients with moderate to severe cancer pain (strong recommendation, moderate certainty evidence).

#### Recommendation statement

1.1 There was no significant difference in relieving cancer pain using different acupuncture techniques (manual acupuncture, electroacupuncture and auricular acupuncture).
1.2 Personalized acupoint selection is suggested. The main acupoints are Hegu (LI4), Taichong (LR3), Ashi-point, Zusanli (ST36), Sanyinjiao (SP6), and Yanglingquan (GB34).
1.3 Manipulation
  Manual acupuncture: The needling consisted of swabbing all acupoints with alcohol. Needles were inserted to a proper depth as determined by standard point locations, and a “De qi” sensation was obtained at all standardized full-body acupuncture points. During the time of acupuncture, the acupuncturist returned to stimulate needles once, to re-elicit the “De qi” sensation. The course is recommended twice a week, 30 min each time for 6 weeks. Due to limited available evidence, it is recommended to consider specific clinical situation in clinical practice.
  Electroacupuncture: Two pairs of electrodes were connected at the needles adjacent to the site of pain with two frequencies (hertz) of electro-stimulation provided by a TENS (Transcutaneous Electric Nerve Stimulation) unit. The course is recommended twice a week for 2 weeks, and then weekly for 6 weeks. The needles were left in place for 30 min each time. Due to limited available evidence, it is recommended to consider specific clinical situation in clinical practice.
  Auricular acupuncture: Use sterile, disposable, semi-permanent needles for 40 min per session, with the needle remaining in the auricle for 3 days. Due to limited available evidence, it is recommended to consider specific clinical situation in the clinical practice.

#### Benefits and harms

Eight randomized controlled trials (RCTs) [28–35], with a total of 530 patients with moderate to severe pain, compared the efficacy of acupuncture and sham acupuncture or wait-list. The results showed that acupuncture could effectively alleviate the intensity of cancer pain (real acupuncture vs. sham acupuncture [28–34], n = 398, NRS score change: MD = − 1.39 points; 95% CI − 2.15 to − 0.63; real acupuncture vs. wait-list control [31, 33, 35], n = 255, NRS score change: MD = − 1.63 points; 95% CI − 2.14 to − 1.13) (see Additional file 1: Appendix S6: Tables S1, S2 and Fig. S1 for summary). No serious treatment-related adverse events were observed. The certainty for evidence was moderate.

Three types of acupuncture including manual acupuncture, electroacupuncture, and auricular acupuncture were identified in included RCTs (Additional file 1: Appendix S6: Tables S3–S5 and Figs. S2–S4). We conducted subgroup analysis based on the types of acupuncture and no statistically significant subgroup effect was found (test of interaction: P = 0.66), indicating that there was no significant difference in relieving cancer pain among different types of acupuncture (Additional file 1: Appendix S6: Fig. S5).

### Clinical question 2: For cancer patients using analgesics for pain control, what are the effects of acupuncture/acupressure when combined with analgesics?

#### Recommendation 2

We suggest a combination treatment with acupuncture/acupressure to reduce pain intensity, decrease opioid dose, and alleviate opioid-related side effects in moderate to severe cancer pain patients who are using analgesics (weak recommendation, low certainty evidence).

#### Recommendation statement

Acupoint selection and manipulation of acupuncture treatment could refer to the recommendation 1. For acupressure, we suggest Shenmen (HT7), Subcortex, and Sympathetic Nerve acupoints. The manipulation is: the patient takes the supine position, and the acupoint skin was routinely disinfected with 75% alcohol. A small piece of tape sticks the cowherb seeds tightly on the acupoints and replaces them daily. Use the index finger and thumb to twist and press the ear points on both sides of the ears, starting from light to heavy. It is suggested that this procedure be performed 3 to 5 times a day in the morning, afternoon, and evening, each time for 3 to 5 min. The pressing force should be an acid bilge feeling in the local point, and 3 days as one course. Due to limited available evidence, the procedure should consider specific clinical practice.

#### Benefits and harms

Three RCTs addressed 224 patients [36–38], and compared a combination of acupuncture and analgesic drugs to analgesic drugs alone. The results suggested that acupuncture combined with analgesics could effectively relieve the intensity of pain (3 studies [36–38], n = 224, NRS score change: MD = − 1.12 points; 95% CI − 2.19 to − 0.06), reduce the use of opioid (1 study [38], n = 60, dose changes: MD = − 22.68 mg/d; 95% CI − 41.70 to − 3.66) (Additional file 1: Appendix S6: Fig. S6).

The systematic review included 3 RCTs with 166 patients with severe cancer pain [39–41] showed that acupressure combined with analgesics could effectively relieve the intensity of pain (3 studies, n = 166, NRS score change: MD = − 1.74 points; 95% CI − 1.98 to − 1.50) and reduce the use of opioid (1 study [40], n = 46, the dose change: MD = − 19.80 mg/d; 95% CI − 24.95 to − 14.65) (Additional file 1: Appendix S6: Fig. S7).

The certainty of evidence for all outcomes above were low (Additional file 1: Appendix S6: Tables S6, S7).

No serious acupuncture-related adverse events were observed. One RCT [39] involving 60 patients with moderate to severe cancer pain showed that with low certainty of evidence, acupressure combined with analgesic could reduce the incidence of nausea and constipation (nausea: OR = 3.04; 95% CI 1.07 to 9.27; constipation: OR = 6.50; 95% CI 1.82 to 23.21).

### Clinical question 3: With regard to breast cancer patients with aromatase inhibitor-induced arthralgia (AIIA), what are the effects of acupuncture treatment?

#### Recommendation 3

We recommend the treatment of acupuncture rather than no treatment to relieve pain in breast cancer patients with aromatase inhibitor-induced arthralgia (strong recommendation, low certainty evidence).

#### Recommendation statement

Acupoint selection and procedure of acupuncture treatment could refer to the recommendation 1.

#### Benefits and harms

The systematic review included 2 RCTs involving 197 patients with moderate to severe cancer pain [31, 33]. The results showed that acupuncture could effectively alleviate the intensity of joint pain caused by aromatase inhibitors (acupuncture vs. wait-list control, NRS score change: MD = − 1.52 points; 95% CI − 2.07 to − 0.97; low certainty) (see Additional file 1: Appendix S6: Table S8 and Fig. S8). No serious treatment-related adverse events of acupuncture were observed.

## Preference and values

Eight [42–49] cross-sectional surveys, that involved 2505 American and Swedish patients, investigated patient’s preference, attitude and barriers. Additional file 1: Appendix S4 presents the details of the study of patient preferences and values, that included search strategies, study screening, summary of included studies, and risk of bias assessment.

Most of cancer patients (ranged from 79 to 97%) thought that acupuncture was worthwhile, important, or effective. Around one third of cancer pain patients (ranged 27% to 42%) preferred acupuncture over medication for pain management. For the patients who received acupuncture treatment, most of them (ranged from 70 to 87%) stated that the course of acupuncture met their expectations; and most of them (ranged 90% to 100%) were willing to receive acupuncture treatment again and were willing to recommend acupuncture to others. The main attitudinal barriers to receive acupuncture treatment included the lack of knowledge of acupuncture, expensive, and needling pain.

## Resources and other considerations

### Safety

The evidence of from the *JAMA Oncology’s* systematic review showed that the adverse events consisted predominantly the skin and subcutaneous tissue disorder or slight pain from the application of treatment to the skin. No dropouts were attributed to adverse effects associated with acupuncture treatment [9]. A study involving 454,920 patients who used acupuncture to treat pain showed that 8% patients experienced mild adverse events, severe side effects occurred in 13 patients (0.003%) [50]. The most frequent mild adverse events included needling pain, hematoma, and bleeding at the point of needle insertion. Severe side reactions were pneumothorax, acute hyper- or hypotensive crisis, erisypelis, and asthma attack [50]. A systematic review indicated that acupuncture performed by trained practitioners using clean needle techniques was a generally safe procedure [51]. A review also showed that due to the low risks of using acupuncture, it could be successfully used for symptom management of cancer patients [52].

### Costs

A systematic review included 8 studies on cost-utility and cost effectiveness of acupuncture for chronic pain showed that the cost per quality-adjusted life-year gained was below the thresholds used by the UK National Institute for Health and Clinical Excellence for “willingness to pay” [53]. A study on the out-of-pocket costs incurred from acupuncture services investigated acupuncture prices of 723 clinics throughout 39 metropolitan regions in the U.S., found that the median cost for a first-time acupuncture visit was $112, and $80 for the follow-up visits [54].

### Policy

Acupuncture is receiving increasing policy support around the world, especially in the area of health insurance.

In USA, acupuncture has been recommended as a non-pharmacologic therapy of the most evidence-based and immediately available choice by the Food and Drug Administration (FDA) as well as the National Academies of Sciences, Engineering, and Medicine (NASEM) in coping with the opioid crisis [55]. Acupuncture was included for the first time in Canada by the province of British Columbia’s Medical Services Plan (MSP) for those who are covered under premium assistance in 2008. This includes patients who are eligible BC residents who have a combined family income of $28,000 or less. Patients have coverage for $23 per visit per year for a maximum combination of 10 sessions from registered acupuncturists and related practitioners [56].

In Australia, the practice of acupuncture has been nationally regulated since 2012. Acupuncture services can be reimbursed by private health funds, and when practiced by medical practitioners, covered by the Australia’s Medicare Benefits Schedule [57]. The Medicare benefit is divided into four levels, including Level A (obvious and straightforward cases and necessary examination, fee: $21.65, benefit 75%), Level B (a consultation lasting less than 20 min for cases that are not obvious or straightforward in relation to one or more health related issues, fee: $37.05, benefit 100%), Level C (a consultation lasting at least 20 min for cases in relation to one or more health related issues, fee: $71.70, benefit 100%), and Level D (a consultation lasting at least 40 min for cases in relation to one or more health related issues, fee: $105.55, benefit 100%).

Chinese medicine in Switzerland is supported by basic and additional medical insurance, which is one of the countries with the greatest support for acupuncture programs [58]. The cost of an acupuncture treatment is about 100–150 Swiss francs, and most patients can be reimbursed 80% from the insurance company. If the doctor performing the treatment is a qualified western doctor, the reimbursement amount will be even greater [59].

In China, National Administration of Traditional Chinese Medicine issued a document, including acupuncture and moxibustion, therapeutic massage, scrapping, cupping and other TCM non-drug diagnosis and treatment technologies into the new rural cooperative medical insurance, 60% reimbursement for township hospitals, 40% for secondary hospitals, and 30% for tertiary hospitals [60].

### Accessibility

Acupuncture has been widely accessible in many countries. The American Academy of Medical Acupuncture (AAMA) has more than 1300 medical doctors trained to offer acupuncture services, and has approved nine programs for medical doctor certification in acupuncture [55, 61]. With the implementation of the Affordable Care Act (ACA), more than 54 million Americans have received acupuncture coverage under the Essential Healthcare Benefit [62]. While the ACA did stipulate acupuncture is a covered modality, the implementation defers to states, which is influenced by both the inertia of the conventional medical establishment and insurance companies’ policies. A survey in 2015 found that the number of licensed acupuncturists in the US was 34,481, up 52.09% from 2004; there were 62 master degree and 10 doctoral degree programs or schools nationwide, and at the same time, 44 states and the District of Columbia that had acupuncture practice laws in place.

In Australia, there are 4921 registered Chinese medicine practitioners in 2020, and 97.9% of them registered as an acupuncturist [63]. In the UK, the majority of acupuncture is performed by health professionals, including doctors, nurses, physiotherapists, midwives and non-medically trained practitioners. All acupuncturists must be members of the appropriate statutory regulatory body, such as General Medical Council for doctors and comply with their standards and codes of conduct. More than a decade ago, the British issued a policy document, as a guideline, which set out a basic, minimum standard of care for acupuncture for cancer pain [64–66]. The British Acupuncture Council (BAcC) has a membership of over 3000 professionally qualified acupuncturists, which is the UK’s largest professional or self-regulatory body for the practice of traditional acupuncture. Each year 2.3 million traditional acupuncture treatments are carried out in the UK, making it one of the most popular complementary therapies [67].

On the other hand, with the development of integrative oncology around the world [68–70], acupuncture has been adopted as part of routine clinical care in the integrative oncology setting [71, 72]. With the wide accessibility of acupuncture, it will continue to play an important role in the field of oncology.

## Discussion

Cancer pain is one of the most common symptoms in cancer patients, especially in advanced cancer patients. However, there were limited effective strategies for pain management for most patients with moderate to severe cancer pain, which significantly reduce the quality of life of patients. Acupuncture can be effective in the management of not only pain, but also symptoms such as insomnia, anxiety, and joint pain [73]. The non-drug treatment and acupuncture therapy were demonstrated to be effective based on available clinical evidence, which offered new avenues for options of managing cancer pain.

This is the first evidence-based guideline for acupuncture in the treatment of cancer pain, and is expected to improve quality of life and care quality for moderate and severe cancer patients. The development of this guideline aims to standardize the clinical practice of acupuncture and ensure its safe and effective application. These guideline recommendations were developed with some strength. Firstly, the evidence was mainly based on a high-quality systematic review published in *JAMA Oncology in 2020*. We conducted meta-analyses to adopt different clinical questions and rated the certainty of evidence with GRADE approach. We focused on the breast cancer patients with aromatase inhibitor-induced arthralgia because of sufficient evidence. Secondly, we recruited 12 international multidisciplinary experts to perform *Delphi* consensus and formulated guideline recommendations by comprehensively considering the magnitude of effect measures, certainty of evidence, patient preferences and values, cost, policy, and accessibility. Thirdly, we considered patient preferences and values based on a systematical review and 2 patient representatives.

However, there were some limitations in this study. Firstly, most of the evidence was of low certainty, and therefore we made weak recommendations. Second, *JAMA Oncology* systematic review searched evidence up to March 31, 2019. We did not update this systematic review because only one additional relevant study [74] was published after their search and could not change the conclusion. Third, the overall evidence is insufficient to recommend acupuncture/acupressure for a specific type of cancer except breast cancer.

Future research should prioritize to address: (1) if the efficacy of acupuncture varies in alleviating cancer pain for different types of cancer; (2) the efficacy of acupressure to relieve cancer pain; (3) the patient preferences, values and attitude barriers of acupuncture for cancer pain from different countries.

## Conclusion

In conclusion, we proposed the first evidence-based guideline recommendations for the management of cancer patients with moderate to severe pain. This guideline will benefit patients with cancer pain using acupuncture treatment. Dissemination and implementation of this guideline are integral components of our goals to address the management of cancer pain. The lack of high-quality evidence limits the strength of the recommendations and highlights the need for additional research.

## Supplementary Information

**Additional file 1: Appendix S1.** The professional field of the steering committee and the expert consensus group. **Appendix S2.** Details of panel members’ declarations of interests. **Appendix S3.** Grading the certainty of evidence and the strength of recommendations. **Appendix S4.** Details of patients preference and values. **Appendix S5.** Details of expert consensus. **Appendix S6.** Summary of findings table.

## Data Availability

All data generated or analyzed during this study are included in Additional files.

## Abbreviations

NRS: Numerical Rating Scale
WHO: World Health Organization
TCM: Traditional Chinese Medicine
GRADE: The Grading of Recommendations Assessment, Development and Evaluation
RIGHT: The Reporting Items for Practice Guidelines in Healthcare
AGREE II: Appraisal of Guidelines for Research and Evaluation II
IOM: Institute of Medicine
CheckUp: The Checklist for the Reporting of Updated Guidelines
TENS: Transcutaneous Electric Nerve Stimulation
RCTs: Randomized Controlled Trials
FDA: Food and Drug Administration
NASEM: The National Academies of Sciences, Engineering, and Medicine
AAMA: The American Academy of Medical Acupuncture
ACA: Affordable Care Act
BAcC: The British Acupuncture Council
