# Supplementary material for: Acupuncture for cancer pain: an evidence-based clinical practice guideline
Source: Chin Med. 2022 Jan 5;17:8. doi: 10.1186/s13020-021-00558-4 (PMC8728906; doi:10.1186/s13020-021-00558-4)
Supplement: Supplementary file 1 — Additional file 1: Appendix S1. The professional field of the steering committee and the expert consensus group. Appendix S2. Details of panel members’ declarations of interests. Appendix S3. Grading the certainty of evidence and the strength of recommendations. Appendix S4. Details of patients preference and values. Appendix S5. Details of expert consensus. Appendix S6. Summary of findings table. [file 13020_2021_558_MOESM1_ESM.docx]

**Data supplement of acupuncture for cancer pain guideline**

Contents

[Appendix S1 The professional field of the steering committee and the expert consensus group 2](#_Toc69849153)

[Appendix S2 Details of panel members’ declarations of interests 3](#_Toc69849154)

[Appendix S3 Grading the Quality of Evidence and the Strength of Recommendations 8](#_Toc69849155)

[Appendix S4 Details of Patients preference and values 9](#_Toc69849156)

[Appendix S5 Details of Expert consensus 16](#_Toc69849161)

[Appendix S6 Summary of Findings Table 17](#_Toc69849162)

## Appendix S1 The professional field of the steering committee and the expert consensus group

| Name | Institution | Expertise |
| --- | --- | --- |
| Steering committee: | | |
| Changli Xue | RMIT University | TCM |
| Haibo Zhang | Guangdong Provincial Hospital of Chinese Medicine | Chinese Medicine Oncology |
| Kehu Yang | EBM Center of Lanzhou University | Methodology |
| Yaolong Chen | EBM Center of Lanzhou University | Methodology |
| Xinfeng Guo | Guangdong Provincial Hospital of Chinese Medicine | Methodology |
| Darong Wu | Guangdong Provincial Hospital of Chinese Medicine | TCM |
| Members of expert consensus group: | | |
| Ka Kit Hui | University of California, Los Angeles | TCM |
| Anthony Lin Zhang | RMIT University | Methodology |
| Darong Wu | Guangdong Provincial Hospital of Chinese Medicine | TCM |
| Nenggui Xu | Guangzhou University of Chinese Medicine | Acupuncture |
| Haiqing Hua | The Affiliated Bayi Hospital of Nanjing University of Chinese Medicine | Oncology |
| Weimin Zhang | Southern Theater Command General Hospital of PLA | Oncology |
| Yufei Yang | Xiyuan Hospital, China Academy of Chinese Medical Sciences | Oncology |
| Bin Xu | Nanjing University of Chinese Medicine | Acupuncture and Massage |
| Fanrong Liang | Chengdu University of TCM | Acupuncture and Massage |
| Baixiao Zhao | Beijing University of Chinese Medicine | Acupuncture and Massage |
| Bing Zhu | China Academy of Chinese Medicine Sciences | Integrated Traditional Chinese and Western Medicine |
| Linpeng Wang | Beijing Hospital of Traditional Chinese Medicine, Capital Medical University | Acupuncture |
| Jinchang Huang | Beijing University of Chinese Medicine Third Affiliated Hospital | Oncology |
| Runsen He | Patient Partners | / |
| Zuodi Pan | Patient Partners | / |

## Appendix S2 Details of panel members’ declarations of interests

- Disclosures Form

**Declaration of Interests for Guideline of acupuncture for cancer pain**

To ensure the highest integrity and public confidence in its activities, the guideline development group (GDG) requires that all participates disclose any circumstances that could give rise to a potential conflict of interest related to the guideline. Please complete this form in time and submit it to the GDG. You must also promptly inform the GDG if there is any change in this information prior to, or during the course of, the guideline.

You must disclose on this Declaration of Interests (DOI) form any financial, professional or other interest relevant to the guideline in which you have been asked to participate in or contribute towards and any interest that could be affected by the outcome of the guideline. You must also declare relevant interests of your immediate family members (see definition below) and, if you are aware of it, relevant interests of other parties with whom you have substantial common interests and which may be perceived as unduly influencing your judgement (e.g., employer, close professional associates, administrative unit or department).

Answering "Yes" to a question on this form does not automatically disqualify you or limit your participation in the guideline. Your answers will be reviewed by the GDG to determine whether you have a conflict of interest relevant to the subject at hand. One of the outcomes listed in the next paragraph can occur depending on the circumstances (e.g., nature and magnitude of the interest, timeframe and duration of the interest).

The GDG may conclude that no potential conflict exists or that the interest is irrelevant or insignificant. If, however, a declared interest is determined to be potentially or clearly significant, one or more of the following three measures for managing the conflict of interest may be applied. The GDG (i) allows full participation, with public disclosure of your interest; (ii) mandates partial exclusion (i.e., you will be excluded from that portion of the guideline related to the declared interest and from the corresponding decision making process); or (iii) mandates total exclusion (i.e., you will not be able to participate in any part of the guideline).

All potentially significant interests will be disclosed to the other participants at the start of the activity and you will be asked if there have been any changes. A summary of all declarations and actions taken to manage any declared interests will be published in resulting reports and work products. If you are unable or unwilling to disclose the details of an interest that may pose a real or perceived conflict, you must disclose that a conflict of interest may exist and the GDG may decide that you be totally recused from the guideline concerned, after consulting with you.

| Name |  | | Institution |  | |
| --- | --- | --- | --- | --- | --- |
| Job Title |  | Duty |  | professional Field |  |
| Tel |  | | Email |  | |
| Address |  | | | | |

**Please answer each of the questions below. If the answer to any of the questions is "Yes", briefly describe the circumstances on the last page of the form.**

"You" refers to yourself and your immediate family members (i.e., spouse (or partner with whom you have a similar close personal relationship) and your children). "Commercial entity" includes any commercial business, an industry association, research institution or other enterprise whose funding is significantly derived from commercial sources with an interest related to the guideline. "Organization" includes a governmental, international or non-profit organization. "Meeting" includes a series or cycle of meetings.

|  | **EMPLOYMENT AND CONSULTING**  Within the past 1 year, have you received remuneration from a commercial entity or other organization with an interest related to the guideline? |  |
| --- | --- | --- |
| 1a | Employment | Yes□ No□ |
| 1b | Consulting, including service as a technical or other advisor | Yes□ No□ |
|  | **RESEARCH SUPPORT**  Within the past 1 year, have you or has your research unit received support from a commercial entity or other organization with an interest related to the guideline? |  |
| 2a | Research support, including grants, collaborations, sponsorships, and other funding | Yes□ No□ |
| 2b | Non-monetary support valued at more than US $1000 overall (include equipment, facilities, research assistants, paid travel to meetings, etc.) Support (including honoraria) for being on a speakers’ bureau, giving speeches or training for a commercial entity or other organization with an interest related to the guideline? | Yes□ No□ |
|  | **INVESTMENT INTERESTS**  Do you have current investments (valued at more than US $5 000 overall) in a commercial entity with an interest related to the guideline? Please also include indirect investments such as a trust or holding company. You may exclude mutual funds, pension funds or similar investments that are broadly diversified and on which you exercise no control. |  |
| 3a | Stocks, bonds, stock options, other securities (e.g., short sales) | Yes□ No□ |
| 3b | Commercial business interests (e.g., proprietorships, partnerships, joint ventures, board memberships, controlling interest in a company) | Yes□ No□ |
|  | **INTELLECTUAL PROPERTY**  Do you have any intellectual property rights that might be enhanced or diminished by the outcome of the guideline? |  |
| 4a | Patents, trademarks, or copyrights (including pending applications) | Yes□ No□ |
| 4b | Proprietary know-how in a substance, technology or process | Yes□ No□ |
|  | **PUBLIC STATEMENTS AND POSITIONS (during the past 1 year)** |  |
| 5a | As part of a regulatory, legislative or judicial process, have you provided an expert opinion or testimony, related to the guideline, for a commercial entity or other organization? | Yes□ No□ |
| 5b | Have you held an office or other position, paid or unpaid, where you represented interests or defended a position related to the guideline? | Yes□ No□ |
|  | **ADDITIONAL INFORMATION** |  |
| 6a | If not already disclosed above, have you worked for the competitor of a product that is the guideline, or will your participation in the guideline enable you to obtain access to a competitor's confidential proprietary information, or create for you a personal, professional, financial or business competitive advantage? | Yes□ No□ |
| 6b | To your knowledge, would the outcome of the guideline benefit or adversely affect interests of others with whom you have substantial common personal, professional, financial or business interests (such as your adult children or siblings, close professional colleagues, administrative unit or department)? | Yes□ No□ |
| 6c | Excluding TCM Recs working group, has any person or entity paid or contributed towards your travel costs in connection with this guideline? | Yes□ No□ |
| 6d | Have you received any payments (other than for travel costs) or honoraria for speaking publicly on this guideline? | Yes□ No□ |
| 6e | Is there any other aspect of your background or present circumstances not addressed above that might be perceived as affecting your objectivity or independence? | Yes□ No□ |

**EXPLANATION OF "YES" RESPONSES: If the answer to any of the above questions is "Yes", check above and briefly describe the circumstances on this page. If you do not describe the nature of an interest or if you do not provide the amount or value involved where relevant, the conflict will be assumed to be significant.**

| Nos. 1-4: Type of interest, question number and category (e.g., Intellectual Property 4.a copyrights) and basic descriptive details. | Name of company, organization, or institution. | Belongs to you, a family member, employer, research unit or other? | Amount of income or value of interest (if not disclosed, is assumed to be significant). | Current interest (or  year ceased). |
| --- | --- | --- | --- | --- |
|  |  |  |  |  |
| Nos. 5-6: Describe the subject, specific circumstances, parties involved, time frame and other relevant details | | | | |
|  | | | | |

**CONSENT TO DISCLOSURE**. By completing and signing this form, I consent to the disclosure of any relevant conflicts to other meeting participants and in the resulting report or work product.

**DECLARATION**. I hereby declare on my honour that the disclosed information is true and complete to the best of my knowledge. Should there be any change to the above information, I will promptly notify the development group and complete a new declaration of interest form that describes the changes. This includes any change that occurs before or during the guideline itself and through the period up to the publication of the final results or completion of the activity concerned.

Date： Signature：

- Disclosures Results

Financial disclosures: No panel members had any financial conflicts of interest to disclose related to this guideline.

Academic disclosures: Charlie Changli Xue, Haibo Zhang and Xinfeng Guo were co-corresponding authors of JAMA Oncology systematic review. Thus, they did not participate in voting the recommendations.

## Appendix S3 Grading the Quality of Evidence and the Strength of Recommendations

- Classification for Certainty and Quality of Evidence Grades

| **Table 1: Quality of Evidence Grades** | |
| --- | --- |
| Grade | Definition |
| High | We are very confident that the true effect lies close to that of the estimate of the effect. |
| Moderate | We are moderately confident in the effect estimate: The true effect is likely to be close to the estimate of the effect, but there is a possibility that it is substantially different. |
| Low | Our confidence in the effect estimate is limited: The true effect may be substantially different from the estimate of the effect. |
| Very Low | We have very little confidence in the effect estimate: The true effect is likely to be substantially different from the estimate of effect. |

- Description for Grading Recommendations

| **Table 2. Implications of strong and weak recommendations for different users of guidelines** | | |
| --- | --- | --- |
|  | Strong Recommendation | Weak Recommendation |
| **For patients** | Most individuals in this situation would want the recommended course of action and only a small proportion would not. | The majority of individuals in this situation would want the suggested course of action, but many would not. |
| **For clinicians** | Most individuals should receive the recommended course of action. Adherence to this recommendation according to the guideline could be used as a quality criterion or performance indicator. Formal decision aids are not likely to be needed to help individuals make decisions consistent with their values and preferences. | Recognize that different choices will be appropriate for different patients, and that you must help each patient arrive at a management decision consistent with her or his values and preferences. Decision aids may well be useful helping individuals making decisions consistent with their values and preferences. Clinicians should expect to spend more time with patients when working towards a decision. |
| **For policy makers** | The recommendation can be adapted as policy in most situations including for the use as performance indicators. | Policy making will require substantial debates and involvement of many stakeholders. Policies are also more likely to vary between regions. Performance indicators would have to focus on the fact that adequate deliberation about the management options has taken place. |

## Appendix S4 Details of Patients preference and values

- Patient Preferences and Values Regarding Acupuncture: search strategy

| **Databases [Platform]** *Searches run* June *22 2020* | **Results** |
| --- | --- |
| Pubmed | 3850 |
| Embase | 4589 |
| Cochrane | 1519 |
| Web of science | 1337 |
| CNKI | 143 |
| WANFANG | 23 |
| VIP | 9 |
| CBM | 152 |
| Subtotal | 11622 |
| -Dupes | 4749 |
| Total | **6873** |

**Pubmed：**

| **1** | **"****Patient Preference*"[Mesh] OR "Patient Participation" [Mesh] OR "patient satisfaction"[Mesh] OR Attitude to Health [Mesh]** | **415141** |
| --- | --- | --- |
| **2** | **"Patient Preference*****"[Title/Abstract] OR "participation preference"[Title/Abstract] OR patient* participation****[Title/Abstract] OR "patient involvement****"[Title/Abstract] OR "patient satisfaction"[Title/Abstract] OR "patient* expectation*"[Title/Abstract] OR "patient accept*"****[Title/Abstract] OR "patient* perspective*"[Title/Abstract] OR "****patient value*"[Title/Abstract] OR "point of view"[Title/Abstract] OR "health state values"[Title/Abstract] OR "patient* view*"[Title/Abstract] OR** **"attitude to health"[Title/Abstract] OR "patient attitude*****"[Title/Abstract] OR "patient decision*"[Title/Abstract] OR "patient choice*"[Title/Abstract] OR "shared decision making"[Title/Abstract] OR "patient centered care"[Title/abstract]** | **157921** |
| **3** | OR/1-2 | **529587** |
| 4 | **"Acupuncture"[Mesh] OR "Acupuncture Therapy****"[Mesh] OR "Acupuncture, Ear"[Mesh] OR "Acupuncture Points"[Mesh] OR "****Acupuncture Analgesia"[Mesh] OR "electroacupuncture"[Mesh]** | 25011 |
| 5 | **Acupuncture[Title/abstract] OR electro-acupuncture[Title/abstract] OR electroacupuncture[Title/abstract] OR "ear acupuncture"[Title/abstract] OR needl*[Title/abstract] OR dry-needl*[Title/abstract] OR auricular[Title/abstract] OR acupressure[Title/abstract]** | 151906 |
| 6 | OR/4-5 | 157067 |
| 7 | 3 AND 6 | 3850 |

**EMBASE：**

| 1 | **'patient preference****'/exp OR 'Patient Participation'/exp OR 'patient satisfaction'/exp OR Attitude to Health/exp** | 286919 |
| --- | --- | --- |
| 2 | **("Patient Preference*" OR "participation preference" OR patient* participation OR "patient involvement" OR "patient satisfaction" OR "patient* expectation*" OR "patient accept*" OR "patient* perspective*" OR "patient value*" OR "point of view" OR "health state values" OR "patient* view*" OR "attitude to health" OR "patient attitude*" OR "patient decision*" OR "patient choice*" OR "shared decision making" OR "patient centered care")**:ti,ab,kw | 222614 |
| 3 | OR/1-2 | 448022 |
| 4 | **'Acupuncture'/exp OR 'auricular acupuncture'/exp OR 'Acupuncture Point'/exp OR 'Acupuncture Analgesia'/exp OR 'electroacupuncture'/exp** | 47234 |
| 5 | **(Acupuncture OR electro-acupuncture OR electroacupuncture OR "ear acupuncture" OR needl* OR dry-needl* OR auricular OR acupressure):ti,ab,kw** | 214346 |
| 6 | OR/4-5 | 227265 |
| 7 | 3 AND 6 | 4589 |

**Cochrane Library:**

| 1 | MeSH descriptor: [Patient Preference] explode all trees **OR** MeSH descriptor: [**Patient Participation**] explode all trees OR MeSH descriptor: [**patient satisfaction**] explode all trees OR MeSH descriptor: [**Attitude to Health**] explode all trees | 34563 |
| --- | --- | --- |
| 2 | **("Patient Preference*" OR "participation preference" OR patient* participation OR "patient involvement" OR "patient satisfaction" OR "patient* expectation*" OR "patient accept*" OR "patient* perspective*" OR "patient value*" OR "point of view" OR "health state values" OR "patient* view*" OR "attitude to health" OR "patient attitude*" OR "patient decision*" OR "patient choice*" OR "shared decision making" OR "patient centered care")**:ti,ab,kw | 53034 |
| 3 | OR /1-2 | 70502 |
| 4 | MeSH descriptor: [Acupuncture] explode all trees OR MeSH descriptor: [**Acupuncture Therapy**] explode all trees OR MeSH descriptor: [**Acupuncture, Ear**] explode all trees OR MeSH descriptor: [**Acupuncture Points**] explode all trees OR MeSH descriptor: [**Acupuncture Analgesia**] explode all trees OR MeSH descriptor: [**electroacupuncture**] explode all trees | 4693 |
| 5 | (Acupuncture OR electro-acupuncture OR **electroacupuncture OR "ear acupuncture"** OR needl* OR dry-needl* OR auricular OR acupressure):ti,ab,kw | 29317 |
| 6 | OR/4-5 | 29420 |
| 7 | 3 AND 6 | 1519 |

**WOS：**

Science Citation Index Expanded (SCI-EXPANDED) –from 1980 to 2020

Social Sciences Citation Index (SSCI) –from 1980 to 2020

| 1 | TS=(Acupuncture OR electro-acupuncture OR **electroacupuncture OR "ear acupuncture"** OR needl* OR dry-needl* OR auricular OR acupressure) | 163633 |
| --- | --- | --- |
| 2 | **TS=("Patient Preference*" OR "participation preference" OR patient* participation OR "patient involvement" OR "patient satisfaction" OR "patient* expectation*" OR "patient accept*" OR "patient* perspective*" OR "patient value*" OR "point of view" OR "health state values" OR "patient* view*" OR "attitude to health" OR "patient attitude*" OR "patient decision*" OR "patient choice*" OR "shared decision making" OR "patient centered care")** | 197191 |
| 3 | 1 AND 2 | 1337 |

**CNKI：**

| 1 | SU="针刺" OR SU="针法" OR SU="针灸" OR SU="耳针" OR SU="电针" OR SU="手针" | 195460 |
| --- | --- | --- |
| 2 | SU="患者偏好" OR SU="患者态度" OR SU="患者满意" OR SU="患者需求" OR SU="患者意愿" OR SU="患者价值观" OR SU="患者观点" OR SU="患者接受" OR SU="患者选择" OR SU="患者期望" OR SU="患者参与" | 75838 |
| 3 | 1 AND 2 | 143 |

**WANFANG：**

| 1 | 题名或关键词:"针刺" OR 题名或关键词:"针法" OR 题名或关键词:"针灸" OR 题名或关键词:"耳针" OR 题名或关键词:"电针" OR 题名或关键词:"手针" | 145869 |
| --- | --- | --- |
| 2 | 题名或关键词:"患者偏好" OR 题名或关键词:"患者态度" OR 题名或关键词:"患者满意" OR 题名或关键词:"患者需求" OR 题名或关键词:"患者意愿" OR 题名或关键词:"患者价值观" OR 题名或关键词:"患者观点" OR 题名或关键词:"患者接受" OR 题名或关键词:"患者选择" OR 题名或关键词:"患者期望" OR 题名或关键词:"患者参与" | 9556 |
| 3 | 1 AND 2 | 23 |

**VIP：**

| 1 | 题名或关键词="针刺" OR 题名或关键词="针法" OR 题名或关键词="针灸" OR 题名或关键词="耳针" OR 题名或关键词="电针" OR 题名或关键词="手针" | 140423 |
| --- | --- | --- |
| 2 | 题名或关键词="患者偏好" OR 题名或关键词="患者态度" OR 题名或关键词="患者价值观" OR 题名或关键词="患者需求" OR 题名或关键词="患者意愿" OR 题名或关键词="患者满意" OR 题名或关键词="患者接受" OR 题名或关键词="患者选择" OR 题名或关键词="患者期望" OR 题名或关键词="患者参与" OR 题名或关键词="患者观点" | 8515 |
| 3 | 1 AND 2 | 9 |

**CBM:**

| 1 | "针灸"[常用字段:智能] OR "针刺"[常用字段:智能] OR "针法"[常用字段:智能] OR "耳针"[常用字段:智能] OR "电针"[常用字段:智能] OR "手针"[常用字段:智能] | 180795 |
| --- | --- | --- |
| 2 | "患者偏好"[常用字段:智能] OR "患者满意"[标题] OR "患者需求"[常用字段:智能] OR "患者价值观"[常用字段:智能] OR "患者意愿"[常用字段:智能] OR "患者态度"[常用字段:智能] OR "患者期望"[常用字段:智能] OR "患者接受"[标题] OR "患者选择"[常用字段:智能] OR "患者参与"[常用字段:智能] OR "患者观点"[常用字段:智能] | 17731 |
| 3 | 1 AND 2 | 152 |

- Flow figure


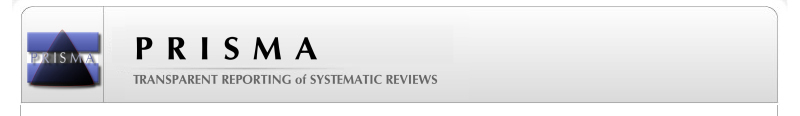
**PRISMA 2009 Flow Diagram**

Records excluded
(n = 6421)

Records screened
(n =6873)

Records after duplicates removed
(n = 6873)

## Identification

## Eligibility

## Included

## Screening

Studies included in quantitative synthesis (meta-analysis)
(n =0)

Studies included in qualitative synthesis
(n = 8)

Full-text articles assessed for eligibility
(n =36)

Full-text articles excluded

(n = 28)

Reason 1: No cancerpain(n=12)

Reason 2: No preference and values of acupuncture(n=13)

Reason 3: No find full-text(n=3)

Additional records identified through other sources
(n = 0)

Records identified through database searching
(n = 11622)

- Outcome

| **Study** | **Country** | **Study methods** | **Sample** | **Cancer types** | **Type of acupuncture** | **Age**  **(Mean,SD)** | **Main findings** |
| --- | --- | --- | --- | --- | --- | --- | --- |
| Johnstone,2002^1^ | USA | Qualitative | 89 | NR | traditional Chinese acupuncture, AA, PENS, and Korean hand and Japanese scalp acupuncture. | 58 (15.3) | About 30% of patients had no response to acupuncture. About the global question regarding acupuncture’s worth: 86% very important, 11% slightly important and 3% unimportant. |
| Liou,2020^2^ | USA | Quantitative | 628 | Breast:31.85%; Thoracic/lung:14.97%; Hematologic:14.97%; Gastrointestinal:12.74%; Head/neck:8.44%; Gynecologic:7.48%; Genito-urinary:5.57%; Other:3.98% | NR | 60.3(11.7) | 31.4% preferred acupuncture for pain management. |
| Mallory,2015^3^ | USA | Quantitative | 20 | breast cancer | NR | 49.50(16.5) | 90% of participants thought that the acupuncture was worthwhile and 95% would have been willing to participate again. 100% participants would recommend the study to others and 70% thought their experience was better than expected. |
| Mao,2014^4^ | USA | Quantitative | 297 | breast cancer | NR | 61.5(9.9) | 49.8% were willing to participate (WTP) in an acupuncture clinical trial. Younger age, higher education, presence of comorbidities, severe joint pain in the past 7 days and greater acupuncture expectancy were significantly associated with greater WTP. |
| Bao,2018^5^ | USA | Quantitative | 592 | breast cancer | NR | 62.8(9.4) | 27% prefer acupuncture for pain treatment. younger age (<60), white race, college-or-above education, higher acupuncture expectancy, natural treatment belief, holistic health belief, social norm scores and lower perceived barrier scores were all statistically significantly associated with preference for acupuncture over medication for pain management. |
| Enblom, 2017^6^ | Sweden | Quantitative | 522 | NR | MA, EA, AA | 64(13.8) | For cancer pain, 89% believed that acupuncture was effective and 42% of them practised it. |
| Frankel, 2014^7^ | USA | Quantitative | 305 | breast:19.7%; prostate:17.7%;  head and neck:18%; gastrointestinal:15.1%; lung:13.4%; another type of cancer:16.1%. | MA | 59.8(12) | 26.4% were likely to use acupuncture during RT if such services were offered at a reasonable price, 46.9% had no knowledge of acupuncture, and 67.6% reported having pain. |
| Garcia, 2014^8^ | USA | Quantitative | 52 | Breast:30.8%; Genitourinaryc:15.4%; Lymphoma/myeloma:15.4%; Melanoma/skin:7.7%; Oro/nasopharyngeal:7.7%; Unknown:7.7%; Brain/spine:5.8%;  Gastrointestinal:3.8%;  Sarcoma:3.8%; Lung:1.9% | AA; acupoints ES | 54(11.9) | 87% stated that the course of acupuncture met their expectations “very well” or “extremely well,” 90% said they were likely to have acupuncture again, 95% said they were likely to recommend acupuncture to others, and 90% said they found the acupuncture to be “useful” or “very useful.” |

NR: not report. PENS: percutaneous electrical nerve stimulation. AA: auricular acupuncture. MA: manual acupuncture; EA: Electroacupuncture.

- Risk of bias

Methodological Limitations Assessments for Qualitative Studies

| **Study** | **Was there a clear statement of the aims of the research?** | **Is a qualitative methodology appropriate?** | **Was the research design appropriate to address the aims of the research?** | **Was the recruitment strategy appropriate to the aims of the research?** | **Was the data collected in a way that addressed the research issue?** | **Has the relationship between researcher and participants been adequately considered?** | **Have ethical issues been taken into consideration?** | **Was the data analysis sufficiently rigorous?** | **Is there a clear statement of findings?** | **How valuable is the research?** | **Overall** |
| --- | --- | --- | --- | --- | --- | --- | --- | --- | --- | --- | --- |
| Johnstone,  2002^1^ | yes | yes | yes | yes | yes | can't tell | no | no | yes | yes | Moderate |

Risk-of-Bias Assessments for Quantitative Studies

| **Study** | **Was an appropriate study sample selected from the sampling frame?** | **Was the response rate sufficiently high to minimize the risk of bias?** | **Did the researchers pilot the measurement techniques on a subset of the target population?** | **Was the instrument validated?** | **Was the instrument reliable?** | **Overall risk of bias** |
| --- | --- | --- | --- | --- | --- | --- |
| Liou,2020^2^ | can't tell | yes | can't tell | yes | yes | low |
| Mallory,2015^3^ | can't tell | no | yes | can't tell | no | high |
| Mao,2014^4^ | can't tell | yes | yes | yes | yes | low |
| Bao,2018^5^ | can't tell | yes | yes | yes | yes | low |
| Enblom, 2017^6^ | can't tell | yes | yes | no | no | high |
| Frankel, 2014^7^ | can't tell | yes | can't tell | no | no | high |
| Garcia, 2014^8^ | can't tell | no | no | yes | no | moderate |

Reference

1. Johnstone PA, Polston GR, Niemtzow RC, et al. Integration of acupuncture into the oncology clinic. Palliat Med. 2002;16(3):235-9.

2. Liou KT, Trevino KM, Meghani SH, et al. Fear of analgesic side effects predicts preference for acupuncture: a cross-sectional study of cancer patients with pain in the USA. Support Care Cancer. 2021;29(1):427-435.

3. Mallory MJ, Croghan KA, Sandhu NP, et al. Acupuncture in the postoperative setting for breast cancer patients: a feasibility study. Am J Chin Med. 2015;43(1):45-56.

4.Mao JJ, Tan T, Li SQ, et al. Attitudes and barriers towards participation in an acupuncture trial among breast cancer patients: a survey study. BMC Complement Altern Med. 2014 ;14:7.

5. Bao T, Li SQ, Dearing JL, et al. Acupuncture versus medication for pain management: a cross-sectional study of breast cancer survivors. Acupunct Med. 2018;36(2):80-87.

6. Enblom A. Patients' and physiotherapists' belief in and use of acupuncture for cancer-related symptoms. Acupunct Med. 2017;35(4):251-258.

7. Frankel E, Garland S, Meghani SH, et al. Patients' Perspectives on Integrating Acupuncture into the Radiation Oncology Setting. Eur J Integr Med. 2014 1;6(5):532-537.

8. Garcia MK, Driver L, Haddad R, et al. Acupuncture for treatment of uncontrolled pain in cancer patients: a pragmatic pilot study. Integr Cancer Ther. 2014 ;13(2):133-40.

## Appendix S5 Details of Expert consensus

We obtained feedback regarding the recommendation from 2 patients and 12 experts.

| **Recommendations** | **Results** |
| --- | --- |
| Recommendation 1: We recommend the treatment of acupuncture patients with moderate to severe cancer pain who have not received other treatments (strong recommendation, moderate certainty evidence). | Agreement: 92.9% |
| One expert suggested need a bit more information on the evidence (and other background information) about “patients with moderate to severe cancer pain who have not received other treatments”. We plan to change to the treatment of acupuncture rather than no treatment. | |
| Recommendation 2: We suggest a combination treatment with acupuncture/acupressure to relieve pain and reduce opioid dose in moderate to severe cancer pain patients who were using analgesics. (weak recommendation, low certainty evidence). | Agreement: 92.9% |
| Recommendation 3: We suggest the treatment of acupuncture rather than no treatment to relieve pain in breast cancer patients with aromatase inhibitor-induced arthralgia. (strong recommendation, low certainty evidence). | Agreement: 71.4% |
| An expert gave an opinion on the strength of the evidence, we considered the quality of the evidence, preferences and values, and other factors, and decided to change to a weak recommendation. | |
| Recommendation 4: For patients with moderate to severe cancer pain, the use of manual acupuncture, auricular acupuncture and electroacupuncture can relieve pain (strong recommendation, low quality evidence) | Agreement: 71.4% |
| Consensus has been reached, but experts have different opinions on whether to retain this recommendation, we decided to add it to the "Recommendation statement". | |
| Recommendation 5.1: Selection acupoints | Agreement: 85.7% |
| Recommendation 5.2: Manipulation | Agreement: 71.4% |
| Recommendation 5.3: Course | Agreement: 71.4% |
| According to one expert's feedback, we added this part to the recommendation statement, and recommended to consider personalized clinical situation in the clinical practice. | |

## Appendix S6 Summary of Findings Table

| **Table S1. Acupuncture compared with sham acupuncture for cancer pain patients** | | | |
| --- | --- | --- | --- |
| Population: Patients with cancer pain | | | |
| Intervention: Acupuncture | | | |
| Control: Sham acupuncture | | | |
| Outcomes | No of Participants（I / C）  （Studies） | Effect size [MD (95%CI)] | Certainty of the Evidence  （GRADE） |
|  |  |  |  |
| pain alleviation（NRS score） | 226/172 (7 studies) | -1.39 points (-2.15, -0.63) | **Moderate** ^a^ |
| CI: Confidence Interval; MD: Mean Difference. | | | |
| a: Downgraded by one level due to heterogeneity (I^2^ = 81%). | | | |

| **Table S2. Acupuncture compared with wait-list control for cancer pain patients** | | | |
| --- | --- | --- | --- |
| Population: Patients with cancer pain | | | |
| Intervention: Acupuncture | | | |
| Control: Wait-list | | | |
| Outcomes | No of Participants（I / C）  （Studies） | Effect size [MD (95%CI)] | Certainty of the Evidence  （GRADE） |
|  |  |  |  |
| pain alleviation（NRS score） | 151/104 (3 studies) | -1.63 points (-2.14, -1.13) | **Moderate** ^a^ |
| CI: Confidence Interval; MD: Mean Difference. | | | |
| a: Downgraded by one level due to risk of bias in lack of blinding. | | | |

**Table S3. Manual acupuncture**

| **Table S3.1. Manual acupuncture compared with sham acupuncture for cancer pain patients** | | | |
| --- | --- | --- | --- |
| Population: Patients with cancer pain | | | |
| Intervention: Manual acupuncture | | | |
| Control: Sham acupuncture | | | |
| Outcomes | No of Participants（I / C）  （Studies） | Effect size [MD (95%CI)] | Certainty of the Evidence  （GRADE） |
|  |  |  |  |
| pain alleviation（NRS score） | 135/85 (3 studies) | -0.88 points (-1.75, -0.01) | **Moderate** ^a^ |
| CI: Confidence Interval; MD: Mean Difference. | | | |
| a: Downgraded by one level due to intersect with minimal important difference (MID)=0.5. | | | |

| **Table S3.2. Manual acupuncture compared with wait-list for cancer pain patients** | | | |
| --- | --- | --- | --- |
| Population: Patients with cancer pain | | | |
| Intervention: Manual acupuncture | | | |
| Control: Wait-list | | | |
| Outcomes | No of Participants（I / C）  （Studies） | Effect size [MD (95%CI)] | Certainty of the Evidence  （GRADE） |
|  |  |  |  |
| pain alleviation（NRS score） | 129/81 (2 studies) | -1.62 points (-2.37, -0.86) | **Low** ^ab^ |
| CI: Confidence Interval; MD: Mean Difference. | | | |
| a: Downgraded by one level due to risk of bias in lack of blinding.  b: Downgraded by one level due to intersect with minimal important difference (MID)=1. | | | |

| **Table S3.3. Manual acupuncture plus analgesic compared with analgesic for cancer pain patients** | | | |
| --- | --- | --- | --- |
| Population: Patients with cancer pain | | | |
| Intervention: Manual acupuncture plus analgesic | | | |
| Control: Analgesic | | | |
| Outcomes | No of Participants（I / C）  （Studies） | Effect size [MD (95%CI)] | Certainty of the Evidence  （GRADE） |
|  |  |  |  |
| pain alleviation（NRS score） | 32/32 (1 study) | -0.83 points (-1.36, -0.30) | **Low** ^ab^ |
| CI: Confidence Interval; MD: Mean Difference. | | | |
| a: Downgraded by one level due to risk of bias in lack of blinding.  b: Downgraded by one level due to intersect with minimal important difference (MID)=0.5. | | | |

**Table S4. Electroacupuncture**

| **Table S4.1 Electroacupuncture compared with sham acupuncture for cancer pain patients** | | | |
| --- | --- | --- | --- |
| Population: Patients with cancer pain | | | |
| Intervention: Electroacupuncture | | | |
| Control: Sham acupuncture | | | |
| Outcomes | No of Participants（I / C）  （Studies） | Effect size [MD (95%CI)] | Certainty of the Evidence  （GRADE） |
|  |  |  |  |
| pain alleviation（NRS score） | 52/52 (2 studies) | -0.84 points (-2.43, 0.75) | **Moderate** ^a^ |
| CI: Confidence Interval; MD: Mean Difference. | | | |
| a: Downgraded by one level due to heterogeneity (I^2^ = 83%). | | | |

| **Table S4.2. Electroacupuncture compared with wait-list control for cancer pain patients** | | | |
| --- | --- | --- | --- |
| Population: Patients with cancer pain | | | |
| Intervention: Electroacupuncture | | | |
| Control: Wait-list | | | |
| Outcomes | No of Participants（I / C）  （Studies） | Effect size [MD (95%CI)] | Certainty of the Evidence  （GRADE） |
|  |  |  |  |
| pain alleviation（NRS score） | 22/23 (1 study) | -2.00 points (-3.11, -0.89) | **Low** ^ab^ |
| CI: Confidence Interval; MD: Mean Difference. | | | |
| a: Downgraded by one level due to risk of bias in lack of blinding.  b: Downgraded by one level due to intersect with minimal important difference (MID)=1. | | | |

| **Table S4.3. Electroacupuncture plus analgesic compared with analgesic for cancer pain patients** | | | |
| --- | --- | --- | --- |
| Population: Patients with cancer pain | | | |
| Intervention: Electroacupuncture plus analgesic | | | |
| Control: Analgesics | | | |
| Outcomes | No of Participants（I / C）  （Studies） | Effect size [MD (95%CI)] | Certainty of the Evidence  （GRADE） |
|  |  |  |  |
| pain alleviation（NRS score） | 80/80 (2 studies) | -1.27 points (-2.93, 0.39) | **Low** ^ab^ |
| CI: Confidence Interval; MD: Mean Difference. | | | |
| a: Downgraded by one level due to risk of bias in lack of blinding.  b: Downgraded by one level due to heterogeneity (I^2^ = 97%). | | | |

**Table S5. Auricular acupuncture**

| **Table S5. Auricular acupuncture compared with sham acupuncture for cancer pain patients** | | | |
| --- | --- | --- | --- |
| Population: Patients with cancer pain | | | |
| Intervention: Auricular acupuncture | | | |
| Control: Sham acupuncture | | | |
| Outcomes | No of Participants（I / C）  （Studies） | Effect size [MD (95%CI)] | Certainty of the Evidence  （GRADE） |
|  |  |  |  |
| pain alleviation（NRS score） | 39/35 (2 studies) | -2.98 points (-5.37, -0.59) | **Low** ^ab^ |
| CI: Confidence Interval; MD: Mean Difference. | | | |
| a: Downgraded by one level due to heterogeneity (I^2^ = 84%).  b: Downgraded by one level due to the small sample size. | | | |

**Table S6. Acupuncture plus analgesic compared with analgesic for cancer pain patients**

| Population: Patients with cancer pain | | | |
| --- | --- | --- | --- |
| Intervention: Acupuncture plus analgesics | | | |
| Control: Analgesics | | | |
| Outcomes | No of Participants（I / C）  （Studies） | Effect size [MD (95%CI)] | Certainty of the Evidence  （GRADE） |
|  |  |  |  |
| pain alleviation（NRS score） | 112/112 (3 studies) | -1.12 points (-2.19, -0.06) | **Low** ^ab^ |
| reduce the dosage of analgesics | 30/30 (1 study) | -22.68mg/d (-41.70, -3.66) | **Low** ^ac^ |
| CI: Confidence Interval; MD: Mean Difference. | | | |
| a: Downgraded by one level due to risk of bias in lack of blinding.  b: Downgraded by one level due to heterogeneity (I^2^ = 95%).  c: Downgraded by one level due to the small sample size. | | | |

**Table S7. Acupressure plus analgesic compared with analgesic for cancer pain patients**

| Population: Patients with cancer pain | | | |
| --- | --- | --- | --- |
| Intervention: Acupressure plus analgesics | | | |
| Control: Analgesics | | | |
| Outcomes | No of Participants（I / C）  （Studies） | Effect size [MD (95%CI)] | Certainty of the Evidence  （GRADE） |
|  |  |  |  |
| pain alleviation（NRS score） | 83/83 (3 studies) | -1.74 points (-1.98, -1.50) | **Low** ^ab^ |
| reduce the dosage of analgesics | 23/23 (1 study) | -19.80mg/d (-24.95, -14.65) | **Low** ^ab^ |
| CI: Confidence Interval; MD: Mean Difference. | | | |
| a: Downgraded by one level due to risk of bias in lack of blinding.  b: Downgraded by one level due to the small sample size. | | | |

**Table S8. Acupuncture compared with wait-list control for cancer pain patients**

| Population: Patients with breast cancer patients who were aromatase inhibitor-induced arthralgia | | | |
| --- | --- | --- | --- |
| Intervention: Acupuncture | | | |
| Control: Wait-list | | | |
| Outcomes | No of Participants（I / C）  （Studies） | Effect size [MD (95%CI)] | Certainty of the Evidence  （GRADE） |
|  |  |  |  |
| pain alleviation（NRS score） | 123/74 (2 studies) | -1.52 points (-2.07, -0.97) | **Low** ^ab^ |
| CI: Confidence Interval; MD: Mean Difference. | | | |
| a: Downgraded by one level due to risk of bias in lack of blinding.  b: Downgraded by one level due to intersect with minimal important difference (MID)=1. | | | |

*
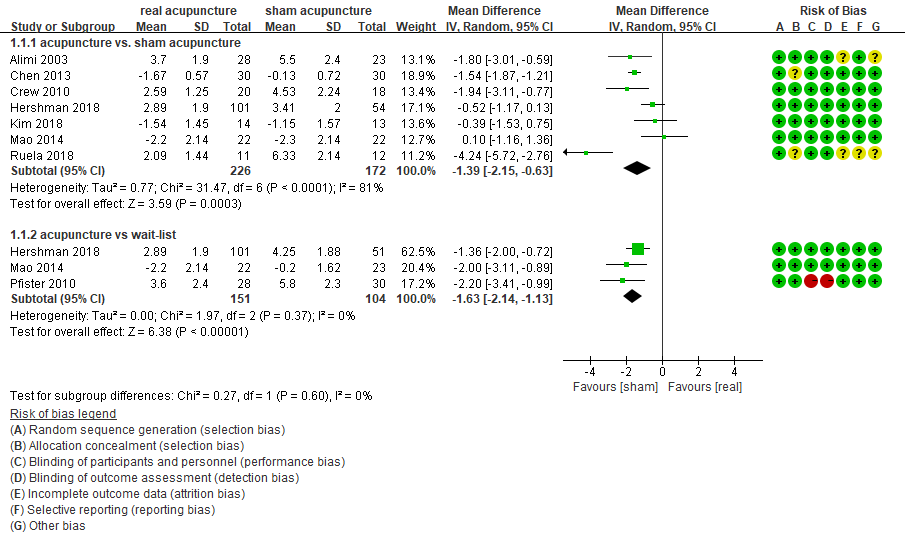
*

**Figure S1. Acupuncture compared with sham acupuncture or wait-list for cancer pain patients**

*
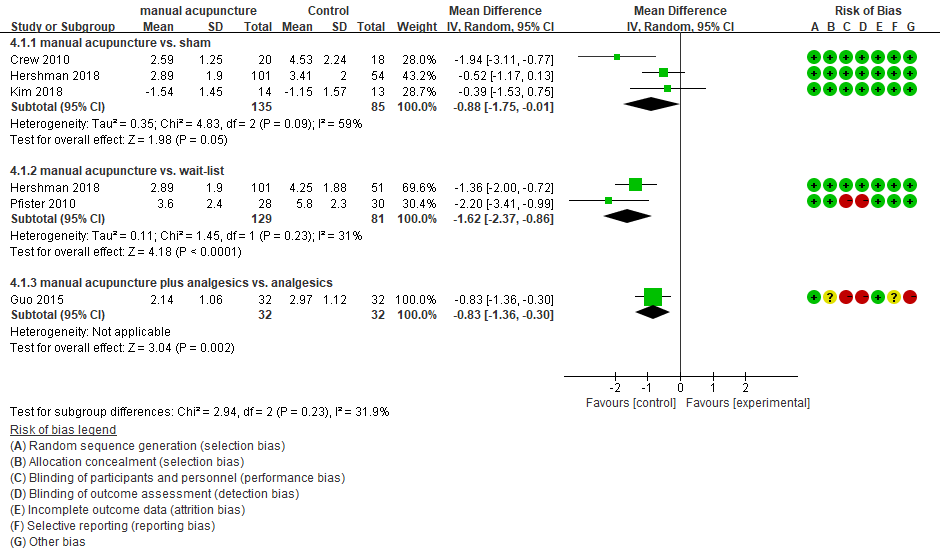
*

**Figure S2. Manual acupuncture for cancer pain patients**

*
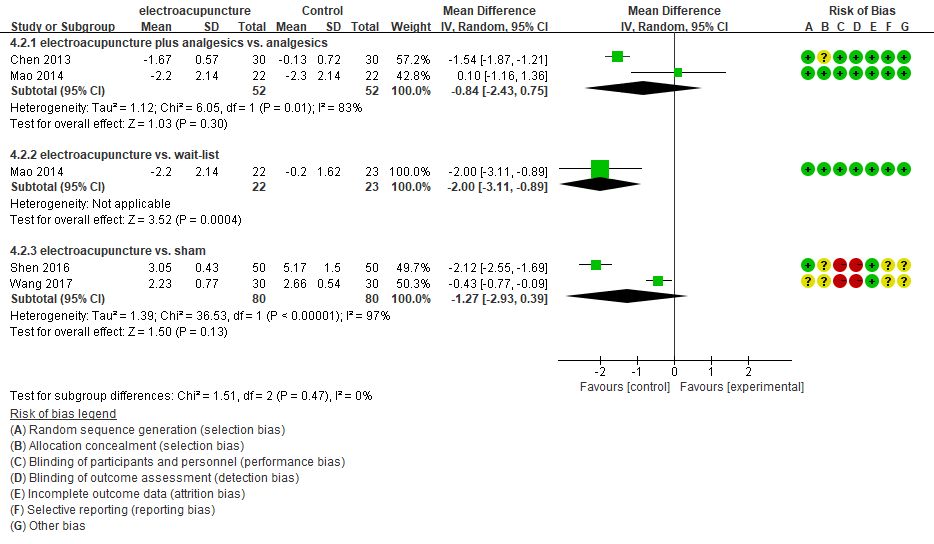
*

**Figure S3. Electroacupuncture for cancer pain patients**

*
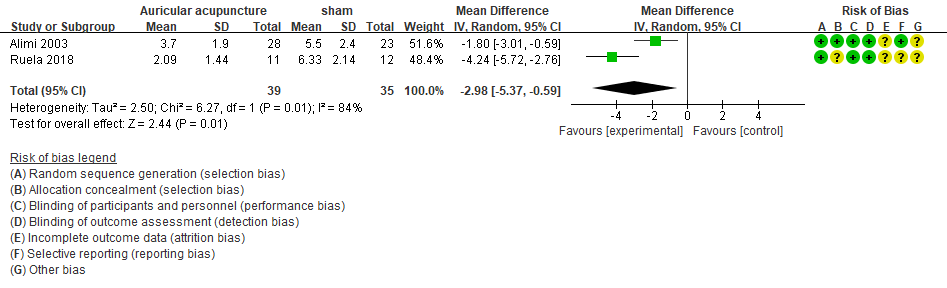
*

**Figure S4. Auricular acupuncture for cancer pain patients**

*
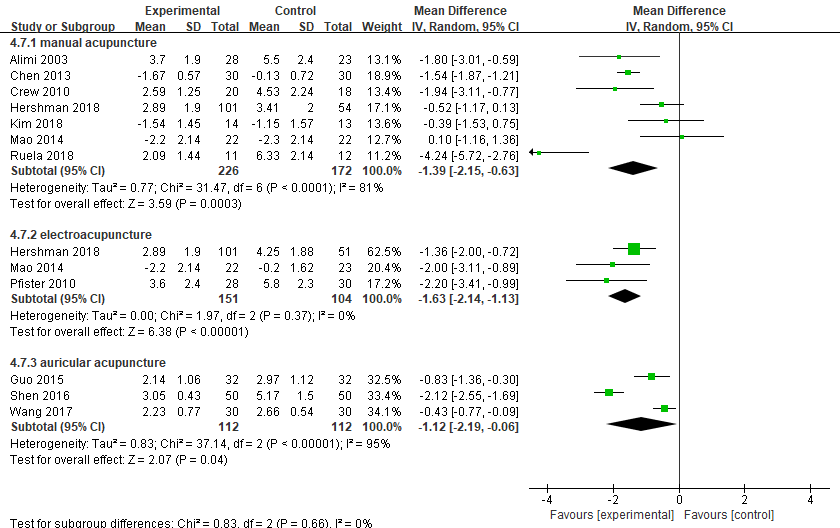
*

**Figure S5. Three types of acupuncture for cancer pain**

**
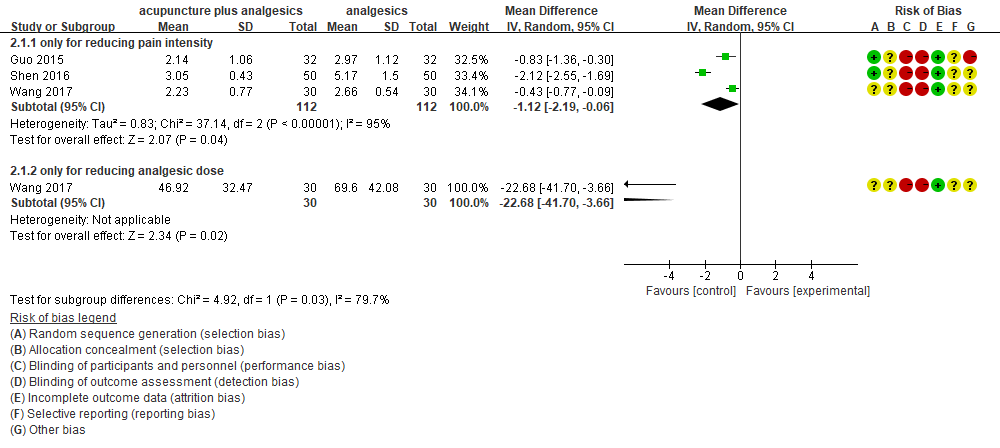
**

**Figure S6. Acupuncture plus analgesic compared with analgesic for cancer pain patients**

*
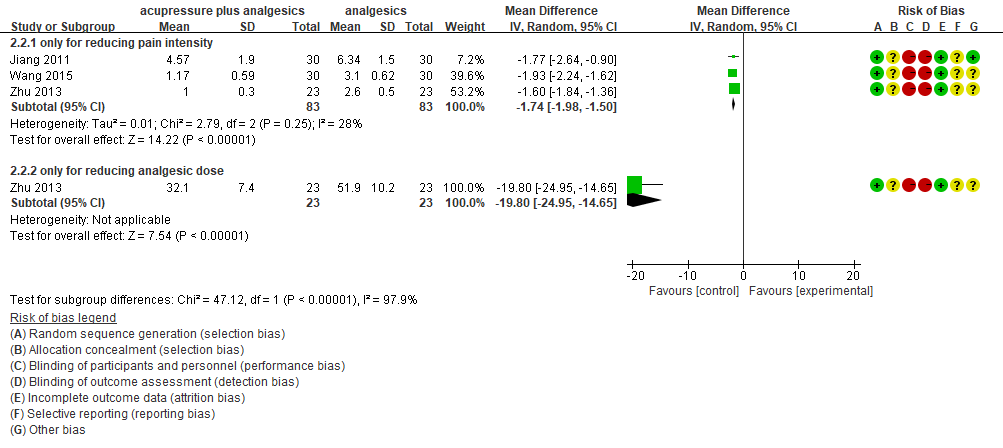
*

**Figure S7. Acupressure plus analgesic compared with analgesic for cancer pain patients**

*
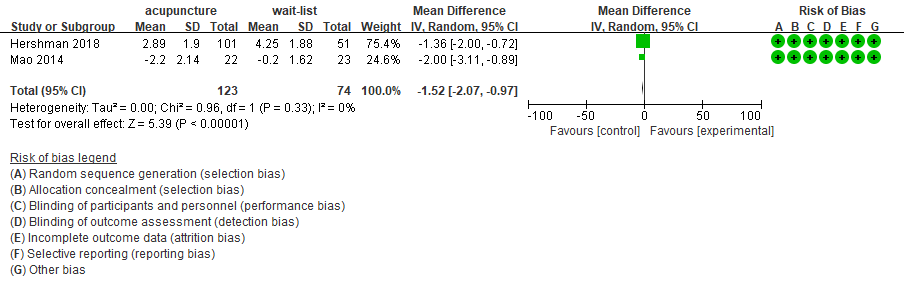
*

**Figure S8. Acupuncture compared with wait-list control for AIIA cancer pain patients**
